# Hypercalcemia and Neurological Symptoms: A Rare Presentation of Hyperfunctioning Parathyroid Adenoma in an Adolescent

**DOI:** 10.3389/fsurg.2022.885188

**Published:** 2022-05-19

**Authors:** Valeria Calcaterra, Gloria Pelizzo, Andreana Pipolo, Giulio Montecamozzo, Valentina Fabiano, Roberta Grazi, Patrizia Carlucci, Gianvincenzo Zuccotti

**Affiliations:** ^1^ Pediatric Department, “Vittore Buzzi” Children’s Hospital, Milan, Italy; ^2^ Pediatric and Adolescent Unit, Department of Internal Medicine, University of Pavia, Pavia, Italy; ^3^ Department of Biomedical and Clinical Sciences “L. Sacco”, University of Milan, Milan, Italy; ^4^ Pediatric Surgery Department, “Vittore Buzzi” Children’s Hospital, Milan, Italy; ^5^ Department of General Surgery, “Luigi Sacco” University Hospital, Milan, Italy

**Keywords:** neuropsychiatric symptoms, hypercalemia, hyperparathyroidism, parathyroid, adenoma children, adolescents

## Abstract

Neuropsychiatric symptoms are rarely described as a manifestation of hyperparathyroidism, especially in children. We describe the case of an adolescent with hypercalcemia related to and hyperfunctioning parathyroid adenoma presenting with acute neuropsychiatric symptoms. A 14-year-old-girl presented into the Emergency Service Department because of an acute onset of marked asthenia, muscle weakness with difficulty in walking, and altered mental status, which included nonsensical speech. No other neurological signs were present. Abdominal, cardiac, and thoracic examination were unremarkable. There was no recent history of trauma or infection. Family history was negative for neurologic disorders. Her past medical history was unremarkable. A head CT scan showed negative results. The laboratory work-up showed elevated levels of calcium level (14.35 mg/dl; nv 9–11 mg/dl), parathyroid hormone (PTH; 184 pg/ml; nv 3.5–36.8 pg/ml), and creatinine (1.23 mg/dl; nv 0.45–0.75 mg/dl). Sodium, potassium, chloride, thyroid function, glycemia, and insulin values were normal. Neck ultrasonography showed a solid, oval, capsulated, hypoechoic neoformation, with discrete vascularization localized to the inferior pole of the right thyroid lobe, referring to parathyroid tissue. Scintigraphy revealed a hyperfunctioning parathyroid tissue at the inferior pole of the right thyroid lobe. Massive intravenous hydration and diuretic therapy were started. The signs and symptoms of hypercalcemia improved after the initiation of therapy. The patient was submitted to right cervicotomy and muscle sparing for the removal of the adenoma of the right superior parathyroid gland. After surgery, a decrease in PTH levels (<4 pg/ml) and calcium levels (9.1 mg/dl) was recorded. During follow-up, calcium values remained stable; a progressive normalization of PTH was obtained. The oral calcium therapy was suspended after 3 months from surgery. No neuropsychiatric symptoms recurred. An evaluation of the serum calcium level is mandatory in children and adolescents with unexplained neurological signs or symptoms, and a check for hyperparathyroidism should be considered.

## Introduction

Hypercalcemia is an infrequent finding in children (1). Through the interplay of parathyroid, renal, and skeletal factors, the serum levels of calcium are maintained in the normal range. Parathyroid hormone (PTH), synthesized and secreted from the parathyroid glands, represents a crucial calciotropic hormone.

The pathogenic mechanisms of hypercalcemia are different and may be age specific, and many have an underlying genetic basis (2). In the differential diagnosis of hypercalcemia, two categories must be considered: PTH-dependent (parathyroid adenoma or carcinoma, familial primary hyperparathyroidism, multiple endocrine neoplasia (MEN) types I, IIa, IV, tertiary hyperparathyroidism) and PTH-independent (drugs, Addison’s disease, pheochromocytoma, malignancies, inborn errors of metabolism, tubular acidosis).

Parathyroid adenoma is responsible for 80%–85% of hyperparathyroidism (3). Primary hyperparathyroidism (PH) is less common in pediatric age than in adult age, with an incidence estimated at only 2–5 in 100,000 and without an apparent sex predilection (4). In the child or adolescent, PH is usually sporadic (65%), and it is due to a single parathyroid adenoma (80%–92%); rarely, parathyroid adenoma occurs as a part of MEN syndromes.

Parathyroid adenoma outgrowth causes the release of more PTHs and leads to dysregulation in calcium and phosphorus levels in the blood. The clinical features of hypercalcemia may be nonspecific and depend upon both the degree of hypercalcemia and the rate of onset of the elevation in the serum calcium concentration: hypercalcemia symptoms may range from an incidental asymptomatic biochemical finding to hypotonia, vomiting, constipation, abdominal pain, lethargy, anorexia, polyuria, polydipsia, poor feeding, and dehydration (1, 2, 5–7). As the calcium concentration increases, symptoms can become more severe: muscle weakness, renal failure, pancreatitis, anxiety, depression, confusion, and stupor can occur. These symptoms are rarely reported in pediatric age (5).

Here, we describe the case of an adolescent with hypercalcemia related to and hyperfunctioning parathyroid adenoma presenting with acute neuropsychiatric symptoms.

## Case Presentation

A 14-year-old-girl presented into the Emergency Service Department because of an acute onset of marked asthenia, massive muscle weakness with difficulty in walking, and altered mental status, which included nonsensical speech.

During the last month, she experienced weight loss, abdominal pain, and a lack of appetite. There was no recent history of trauma or infection. Family history was negative for neurologic disorders. Her past medical history was unremarkable.

On admission, temperature, blood pressure, heart, and respiratory rate were normal. Weight: 42 kg (−1 SDS), Height: 135 cm (−3.9 SDS), and BMI: 24.01 kg/m^2^ (+1 SDS). Physical examination showed marked hyposthenia with difficulty in walking, bradykinesia, and slurred speech. No other neurological signs were present. Abdominal, cardiac, and thoracic examination were unremarkable.

A contrast-enhanced head CT scan showed negative results. The initial laboratory work-up showed elevated levels of calcium level (14.35 mg/dl; normal range: 9–11 mg/dl), PTH (184 pg/ml; normal range: 3.5–36.8 pg/ml), and creatinine (1.23 mg/dl, normal range: 0.45–0.75 mg/dl). Sodium, potassium, chloride, thyroid function, glycemia, and insulin values were normal. Toxicology report was negative.

Serial ECG recordings showed a slightly shortened QT interval and EEG was normal.

**Figure 1:**
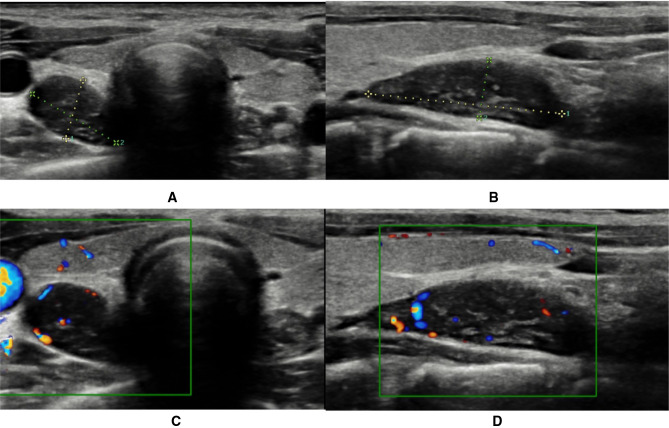
Ultrasound (**A–B**) and color Doppler ultrasound (**C–D**) images of parathyroid lesion.

**Figure 2:**
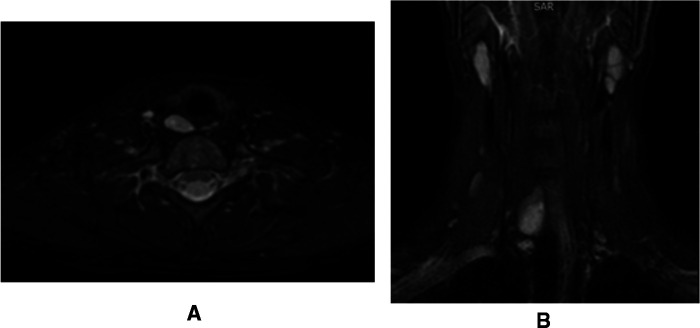
Parathyroid adenoma visualization on magnetic resonance imaging. (**A**) Transversal projection; (**B**) Coronal projection.

Neck ultrasonography showed a solid, oval, capsulated, hypoechoic neoformation (21 × 7 mm), with discrete vascularization localized to the inferior pole of the right thyroid lobe, referring to parathyroid tissue (Figure 1). Magnetic resonance imaging (MRI) showed a solid, oval formation at the lower pole of the right thyroid lobe with early vascularization compatible with the suspicion of parathyroid neoformation (Figure 2).

Planar scintigraphy revealed a hyperfunctioning parathyroid tissue at the inferior pole of the right thyroid lobe.

Massive hydration with intravenous fluids and diuretic therapy with furosemide were started upon admission to the Pediatric Department. The signs and symptoms of hypercalcemia rapidly improved after the initiation of therapy. During hospitalization, calcium levels improved but remained stably high.

**Figure 3:**
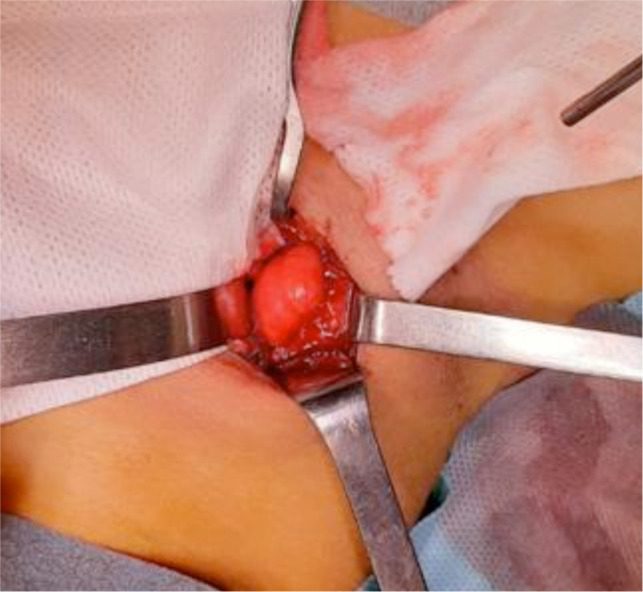
Intraoperative image of the parathyroid adenoma.

Parathyroidectomy was finally performed. The patient was submitted to right cervicotomy and muscle sparing for the removal of a voluminous adenoma of the right superior parathyroid gland (Figure 3). Cautious blunt detachment of the pathological gland until identification of the vascular peduncle was performed. The lesion was found originating at the point of entry of the recurrent nerve into the larynx and to be adhering to the pretracheal region. Intraoperative pathologic evaluation confirmed the diagnosis of adenoma of the parathyroid with main oxyphil cell aspects.

No perioperative complications occurred, with a decrease in PTH levels (<4 pg/ml) and calcium levels (9.1 mg/dl).

The patient was discharged after 3 days from the intervention. As reported in Table 1, at discharge, calcium levels were normal and PTH levels were low; preventive calcium supplementation therapy was started upon discharge.

**Table 1:** Serum calcium and PTH levels before and after surgery.

Parameters	Preoperative	Postoperative monitoring					
		Day 1	Day 2	Day 3	Day 13	1 month	3 months
Calcium (9–11 mg/dl)	14.35	13.6	10.2	9.5	9.8	10.3	9.8
Ca^+^ (4.7–5.2 mg/dl)					5.18	5.26	5.03
PTH (3.5–36.8 pg/ml)	184	11.2	<4	<4	32	60^a^	63^a^
Calcium supplementation	–	–	–	Yes	Yes	Yes	No

*PTH, Parathyroid hormone*.

*^a^vn 18.5–88 pg/ml*.

During follow-up, calcium values remained stable; a progressive normalization of PTH was obtained. The oral calcium therapy was suspended after 3 months from surgery.

During follow-up, no recurrence of neurological or psychiatric symptoms was noted.

The genetic make-up with the next-generation sequency-based gene panel test (NGS—Custom Panel Enrichment and Nextera Flex Enrichment) was performed to exclude the genetic forms of PH. No significant alterations in the coding sequences of the examined genes (*AIP*, *APC*, *BRAF*, *CASR*, *CDC73*, *CDKN1B*, *DICER1*, *EPAS1*, *FXN*, *GCM2*, *GDNF*, *GNAS*, *GPR101*, *HAPB2*, *HRAS*, *KIF1B*, *KIT*, *KRAS*, *MAP2K5*, *MAX*, *MEN1*, *NF1*, *NRAS*, *PARP4*, *PRKAR1A*, *PTEN*, *RET*, *SDHA*, *SDHAF2*, *SDNB*, *SDNC*, *SDHD*, *TMEM127*, *VHL*) were detected.

## Discussion

We reported a rare presentation of PH in an adolescent in which neurological symptoms occurred as a manifestation of hypercalcemia.

In pediatrics, PH is most commonly the presenting manifestation of a single parathyroid adenoma. PH can also be an autosomal dominant genetic disorder that is typically associated with multigland hyperplasia. Our patient had no family history of parathyroid disease or other endocrinopathies, and, consequently, the suspicion for MEN disease was low. However, sporadic MEN syndrome can still be a possibility, where *de novo* mutations in *MEN1* are found in 10% of MEN1 patients; therefore, especially in younger age groups, genetic testing is important to exclude MEN and/or other genetic forms of hypercalcemia.

Neuropsychiatric symptoms are rarely described as a manifestation of hyperparathyroidism, especially in children. The most common symptoms are anxiety, depression, and cognitive dysfunction. More severe symptoms, including lethargy, confusion, stupor, and coma, may occur in patients with severe hypercalcemia (8); these symptoms are more likely to occur in older adults and in those with rapidly rising calcium concentrations (9). Less information is available in the literature on pediatric age. Minelli et al. (10) described the case of an adolescent with neuropsychiatric symptoms caused by hypercalcemia due to a mediastinal parathyroid adenoma. Babar et al. (11) reported the case of a 17-year-old adolescent male, who presented with an acute psychosis coinciding with severe hypercalcemia caused by a benign parathyroid adenoma. Teodoriu et al. (12) described the case of a 16-year-old adolescent girl, who presented with a disturbance of affectivity and mild memory impairment caused by hypercalcemia due to a parathyroid adenoma. As in our case, symptoms vanished after treatment, supporting the fact that neuropsychiatric symptoms are believed to represent a direct effect of hypercalcemia on the central nervous system.

The pathogenesis of neuropsychiatric symptoms is not completely understood: high calcium levels can be a catalyst for neuronal demise, possibly due to glutaminergic excitotoxicity and dopaminergic and serotonergic dysfunction (13, 14). Calcium appears to play an important role in causing changes in monoamine metabolism, determining the modification of dopaminergic and cholinergic metabolism and release at several neuroregulatory stages, and it may affect behavior and mood in some patients (15, 16). An alternate hypothesis is that the psychiatric symptoms may be related to a number of factors like premorbid adjustment and sociocultural influences (17). Also, severe hypercalcemia inhibits neuromuscular and myocardial depolarization, leading to muscle weakness and arrhythmias.

Patients with severe (calcium > 14 mg/dl) or symptomatic hypercalcemia usually require saline hydration as initial therapy, adjusted to maintain the urine output at 100 to 150 ml/h (18). Concurrent treatment with bisphosphonates with or without calcitonin may be required to treat moderate-to-severe hypercalcemia. Administration of a loop diuretic is not routinely recommended (19). Medical treatment may improve the neurological symptoms related to hypercalcemia; however, surgery remains the cornerstone of the management of PHPT.

This case shows that an evaluation of the serum calcium level is mandatory in children and adolescents with unexplained neurological signs or symptoms, and a check for hyperparathyroidism should be considered. Medical therapy and surgery has been associated with a resolution of the hypercalcemia and neuropsychological symptoms.

## Data Availability

The raw data supporting the conclusions of this article will be made available by the authors, without undue reservation.
